# 4-(3-Fluoro-4-methyl­anilino)-2-methyl­idene-4-oxo­butanoic acid

**DOI:** 10.1107/S160053681302998X

**Published:** 2013-11-09

**Authors:** Prakash S. Nayak, B. Narayana, Jerry P. Jasinski, H. S. Yathirajan, Manpreet Kaur

**Affiliations:** a Department of Studies in Chemistry, Mangalore University, Mangalagangotri 574 199, India; b Department of Chemistry, Keene State College, 229 Main Street, Keene, NH 03435-2001, USA; c Department of Studies in Chemistry, University of Mysore, Manasagangotri, Mysore 570 006, India

## Abstract

The title compound, C_12_H_12_FNO_3_, crystallizes with two independent mol­ecules (*A* and *B*) in the asymmetric unit. The dihedral angle between the mean planes of the 3-fluoro-4-methyl­phenyl ring and the oxo­amine group is 25.7 (7)° in mol­ecule *A* and 71.3 (7)° in mol­ecule *B*, while the mean plane of the 2-methyl­idene-4-oxo­butanoic acid group is twisted by 76.2 (1)° from that of the oxo­amine group in mol­ecule *A* and by 76.2 (4)° in mol­ecule *B*. In the crystal, N—H⋯O and O—H⋯O hydrogen bonds [the latter forming an *R*_2_^2^(8) graph-set motif] link the mol­ecules into a two-dimensional network parallel to the *ac* plane.

## Related literature

For properties of itaconic anhydride polymers, see: Oishi (1980 ▶); Urzua *et al.* (1998 ▶). For derivatives of itaconic anhydride, see: Katla *et al.* (2011 ▶); Shetgiri & Nayak (2005 ▶); Hanoon (2011 ▶); Nayak *et al.* (2013 ▶). For standard bond lengths, see: Allen *et al.* (1987 ▶).
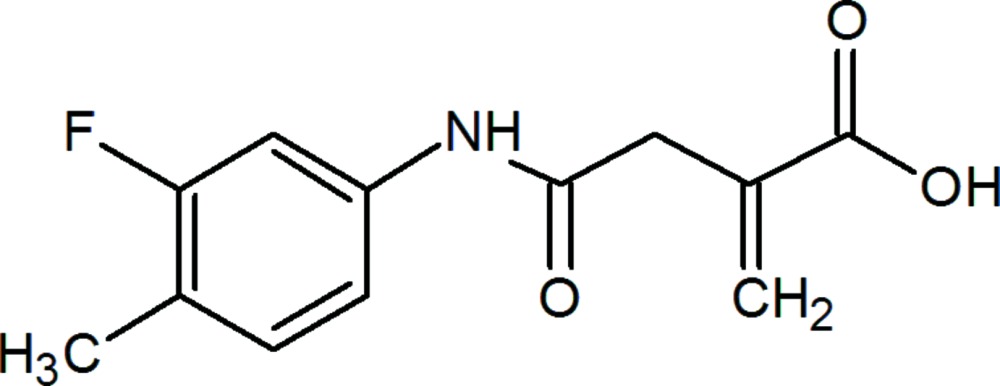


## Experimental

### 

#### Crystal data

C_12_H_12_FNO_3_
*M* *_r_* = 237.23
Triclinic, 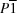

*a* = 6.3368 (3) Å
*b* = 8.2642 (4) Å
*c* = 21.0277 (11) Å
α = 84.057 (4)°
β = 89.798 (4)°
γ = 86.062 (4)°
*V* = 1092.69 (9) Å^3^
*Z* = 4
Mo *K*α radiation
μ = 0.12 mm^−1^
*T* = 173 K
0.38 × 0.32 × 0.16 mm

#### Data collection

Agilent Xcalibur (Eos, Gemini) diffractometer
Absorption correction: multi-scan (*CrysAlis PRO* and *CrysAlis RED*; Agilent, 2012 ▶) *T*_min_ = 0.673, *T*_max_ = 1.000
13094 measured reflections
7221 independent reflections
4872 reflections with *I* > 2σ(*I*)
*R*_int_ = 0.032

#### Refinement

*R*[*F* ^2^ > 2σ(*F* ^2^)] = 0.081
*wR*(*F* ^2^) = 0.221
*S* = 1.06
7221 reflections
327 parameters
H atoms treated by a mixture of independent and constrained refinement
Δρ_max_ = 0.68 e Å^−3^
Δρ_min_ = −0.28 e Å^−3^

### 

Data collection: *CrysAlis PRO* (Agilent, 2012 ▶); cell refinement: *CrysAlis PRO*; data reduction: *CrysAlis RED* (Agilent, 2012 ▶); program(s) used to solve structure: *SUPERFLIP* (Palatinus & Chapuis, 2007 ▶); program(s) used to refine structure: *SHELXL2013* (Sheldrick, 2008 ▶); molecular graphics: *OLEX2* (Dolomanov *et al.*, 2009 ▶); software used to prepare material for publication: *OLEX2*.

## Supplementary Material

Crystal structure: contains datablock(s) I. DOI: 10.1107/S160053681302998X/bv2227sup1.cif

Structure factors: contains datablock(s) I. DOI: 10.1107/S160053681302998X/bv2227Isup2.hkl

Supplementary material file. DOI: 10.1107/S160053681302998X/bv2227Isup3.cml

Additional supplementary materials:  crystallographic information; 3D view; checkCIF report

## Figures and Tables

**Table 1:** Hydrogen-bond geometry (Å, °)

*D*—H⋯*A*	*D*—H	H⋯*A*	*D*⋯*A*	*D*—H⋯*A*
O2*A*—H2*A*⋯O3*A* ^i^	0.82	1.84	2.658 (2)	174
O2*B*—H2*B*⋯O3*B* ^ii^	0.82	1.85	2.667 (2)	176
N1*A*—H1*A*⋯O1*B* ^iii^	0.86	2.10	2.948 (2)	168
N1*B*—H1*B*⋯O1*A* ^iv^	0.86	2.10	2.884 (2)	151

Symmetry codes: (i) 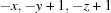; (ii) 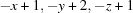; (iii) 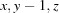; (iv) 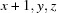.

**Fig. 1.**
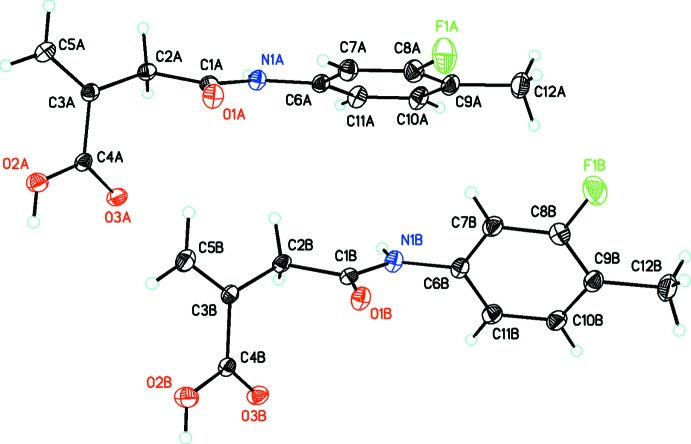
ORTEP drawing of (I), C12H12FNO3, showing the labeling scheme with two molecules (A & B) in the asymmetric unit and 30% probability displacement ellipsoids.

**Fig. 2.**
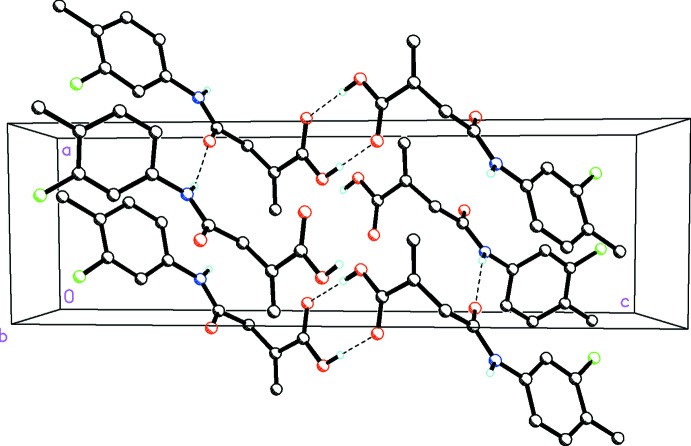
Molecular packing for (I) viewed along the b axis. Dashed lines indicate N—H···O hydrogen bonds and O—H···O R22(8) graph set motif hydrogen bonds which link the molecules into a 2-D network along the ac plane. H atoms not involved in hydrogen bonding have been removed for clarity.

**Fig. 3.**
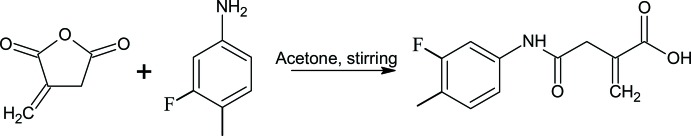
Synthesis scheme of (I).

## supplementary crystallographic information

### 1. Comment

Itaconic anhydride (ITA) is a monomer obtained from renewable resources. Copolymers containing both hydrophilic and hydrophobic segments are drawing considerable attention because of their possible use in biological systems. N-Substituted itaconamic acids are strongly amphiphilic molecules. Itaconic anhydride is more reactive than maleic anhydride and is an alternative monomer for introducing polar functionality into polymers (Oishi, 1980; Urzua *et al.*, 1998). The basic skeleton of itaconic anhydride is useful for the synthesis of various biodynamic cyclic derivatives such as imides (Shetgiri & Nayak, 2005), pyridazine (Katla *et al.*, 2011), oxazepine (Hanoon, 2011) and oxobutanoic acid (Nayak *et al.*, 2013) derivatives. Hence in view of the importance of anhydride derivatives, the crystal structure of the title compound, C_12_H_12_NO_3_F, (I), is reported here.

The title compound, (I), crystallizes with two independent molecules (A & B) in the asymmetric unit (Fig. 1). The dihedral angle between the mean planes of the 3-fluoro-4-methylphenyl ring and the oxo-amine group is 25.7 (7)° (A) and 71.3 (7)Å (B), while the mean plane of the 2-methylidene-4-oxobutanoic acid group is twisted by 76.2 (1)Å (A) and 76.2 (4)Å (B) from that of the oxo-amine group. In the crystal, N—H···O hydrogen bonds and O—H···O R_2_^2^(8) graph set motif hydrogen bonds link the molecules into a 2-D network along the *ac* plane (Fig. 2) and influence crystal packing.

### 2. Experimental

Itaconic anhydride (0.112 g, 1 mmol) dissolved in a 30 ml acetone and it was stirred at ambient temperature and 3-fluoro-4-methyl aniline (0.125 g, 1 mmol) was added portion wise over 30 mins (Fig. 3) The mixture turned into yellow slurry. After stirring 1.5hrs, the slurry was filtered. The solid was washed with acetone and dried to give title compound (I). Single crystals were grown from methanol by the slow evaporation method and used as such for x-ray studies.(M.P.: 414-416 K).

### 3. Refinement

All of the H atoms were placed in their calculated positions and then refined using the riding model with Atom—H lengths of 0.93Å (CH), 0.97Å (CH_2_), 0.96Å (CH_3_), 0.82Å (OH) or 0.86Å (NH). Isotropic displacement parameters for these atoms were set to 1.2 (CH, CH_2_, NH) or 1.5 (CH_3_, OH) times *U*_eq_ of the parent atom. Idealised Me and tetrahedral OH refined as rotating groups.

### Crystal data

**Table:**

C_12_H_12_FNO_3_	*Z* = 4
*M**_r_* = 237.23	*F*(000) = 496
Triclinic, *P*1	*D*_x_ = 1.442 Mg m^−^^3^
*a* = 6.3368 (3) Å	Mo *K*α radiation, λ = 0.7107 Å
*b* = 8.2642 (4) Å	Cell parameters from 3167 reflections
*c* = 21.0277 (11) Å	θ = 3.3–32.7°
α = 84.057 (4)°	µ = 0.12 mm^−^^1^
β = 89.798 (4)°	*T* = 173 K
γ = 86.062 (4)°	Irregular, colourless
*V* = 1092.69 (9) Å^3^	0.38 × 0.32 × 0.16 mm

### Data collection

**Table:**

Agilent Xcalibur (Eos, Gemini) diffractometer	7221 independent reflections
Radiation source: Enhance (Mo) X-ray Source	4872 reflections with *I* > 2σ(*I*)
Graphite monochromator	*R*_int_ = 0.032
Detector resolution: 16.0416 pixels mm^-1^	θ_max_ = 32.8°, θ_min_ = 3.3°
ω scans	*h* = −9→9
Absorption correction: multi-scan (*CrysAlis PRO* and *CrysAlis RED*; Agilent, 2012)	*k* = −11→12
*T*_min_ = 0.673, *T*_max_ = 1.000	*l* = −30→31
13094 measured reflections	

### Refinement

**Table:**

Refinement on *F*^2^	Primary atom site location: structure-invariant direct methods
Least-squares matrix: full	Hydrogen site location: mixed
*R*[*F*^2^ > 2σ(*F*^2^)] = 0.081	H atoms treated by a mixture of independent and constrained refinement
*wR*(*F*^2^) = 0.221	*w* = 1/[σ^2^(*F*_o_^2^) + (0.0895*P*)^2^ + 0.6971*P*] where *P* = (*F*_o_^2^ + 2*F*_c_^2^)/3
*S* = 1.06	(Δ/σ)_max_ < 0.001
7221 reflections	Δρ_max_ = 0.68 e Å^−^^3^
327 parameters	Δρ_min_ = −0.28 e Å^−^^3^
0 restraints	

### Special details

**Table:**

Geometry. All esds (except the esd in the dihedral angle between two l.s. planes) are estimated using the full covariance matrix. The cell esds are taken into account individually in the estimation of esds in distances, angles and torsion angles; correlations between esds in cell parameters are only used when they are defined by crystal symmetry. An approximate (isotropic) treatment of cell esds is used for estimating esds involving l.s. planes.

### Fractional atomic coordinates and isotropic or equivalent isotropic displacement parameters (Å^2^)

**Table:**

	*x*	*y*	*z*	*U*_iso_*/*U*_eq_	
F1A	0.2264 (3)	0.7138 (2)	0.08603 (8)	0.0598 (5)	
O1A	−0.0578 (3)	0.49102 (19)	0.28774 (8)	0.0317 (4)	
O2A	−0.2598 (3)	0.4663 (2)	0.46934 (8)	0.0326 (4)	
H2A	−0.1978	0.5069	0.4973	0.049*	
O3A	0.0704 (2)	0.3813 (2)	0.44375 (8)	0.0301 (3)	
N1A	0.1954 (3)	0.3003 (2)	0.26309 (9)	0.0267 (4)	
H1A	0.2466	0.2026	0.2745	0.032*	
C1A	0.0240 (3)	0.3514 (2)	0.29537 (9)	0.0223 (4)	
C2A	−0.0635 (3)	0.2194 (2)	0.34215 (10)	0.0253 (4)	
H2AA	−0.1345	0.1443	0.3184	0.030*	
H2AB	0.0528	0.1584	0.3656	0.030*	
C3A	−0.2160 (3)	0.2886 (2)	0.38861 (10)	0.0239 (4)	
C4A	−0.1221 (3)	0.3827 (2)	0.43641 (10)	0.0236 (4)	
C5A	−0.4195 (4)	0.2636 (3)	0.38952 (12)	0.0324 (5)	
H5AA	−0.518 (5)	0.312 (4)	0.4188 (14)	0.038 (8)*	
H5AB	−0.483 (5)	0.204 (4)	0.3585 (15)	0.049 (9)*	
C6A	0.3022 (4)	0.3877 (3)	0.21259 (10)	0.0266 (4)	
C7A	0.2073 (4)	0.5190 (3)	0.17415 (11)	0.0310 (5)	
H7A	0.0701	0.5593	0.1820	0.037*	
C8A	0.3231 (4)	0.5879 (3)	0.12384 (11)	0.0349 (5)	
C9A	0.5269 (4)	0.5377 (3)	0.10906 (12)	0.0351 (5)	
C10A	0.6179 (4)	0.4087 (3)	0.14927 (13)	0.0388 (6)	
H10A	0.7570	0.3716	0.1421	0.047*	
C11A	0.5091 (4)	0.3328 (3)	0.19983 (12)	0.0337 (5)	
H11A	0.5743	0.2450	0.2253	0.040*	
C12A	0.6457 (5)	0.6181 (4)	0.05352 (13)	0.0477 (7)	
H12D	0.5574	0.7058	0.0320	0.072*	
H12E	0.6843	0.5394	0.0242	0.072*	
H12F	0.7712	0.6602	0.0689	0.072*	
F1B	0.6414 (3)	1.0662 (3)	0.05324 (7)	0.0555 (5)	
O1B	0.4220 (3)	0.97672 (18)	0.28923 (8)	0.0288 (3)	
O2B	0.2374 (3)	0.9727 (2)	0.47086 (8)	0.0350 (4)	
H2B	0.3050	1.0169	0.4965	0.053*	
O3B	0.5598 (2)	0.8739 (2)	0.44475 (8)	0.0318 (4)	
N1B	0.6529 (3)	0.7751 (2)	0.26178 (9)	0.0278 (4)	
H1B	0.6969	0.6743	0.2696	0.033*	
C1B	0.4934 (3)	0.8347 (2)	0.29677 (9)	0.0214 (4)	
C2B	0.4038 (3)	0.7091 (2)	0.34588 (10)	0.0248 (4)	
H2BA	0.3250	0.6348	0.3240	0.030*	
H2BB	0.5194	0.6460	0.3689	0.030*	
C3B	0.2614 (3)	0.7880 (2)	0.39254 (9)	0.0234 (4)	
C4B	0.3671 (3)	0.8823 (2)	0.43834 (9)	0.0233 (4)	
C5B	0.0546 (4)	0.7733 (3)	0.39513 (12)	0.0321 (5)	
H5BA	−0.030 (4)	0.831 (3)	0.4239 (12)	0.029 (7)*	
H5BB	−0.013 (5)	0.715 (4)	0.3643 (14)	0.040 (8)*	
C6B	0.7520 (3)	0.8736 (3)	0.21197 (10)	0.0260 (4)	
C7B	0.6465 (4)	0.9236 (3)	0.15526 (11)	0.0304 (5)	
H7B	0.5093	0.8950	0.1489	0.036*	
C8B	0.7488 (4)	1.0169 (3)	0.10828 (11)	0.0334 (5)	
C9B	0.9529 (4)	1.0626 (3)	0.11392 (11)	0.0324 (5)	
C10B	1.0547 (4)	1.0105 (3)	0.17149 (11)	0.0340 (5)	
H10B	1.1919	1.0394	0.1776	0.041*	
C11B	0.9578 (4)	0.9164 (3)	0.22030 (11)	0.0308 (5)	
H11B	1.0301	0.8823	0.2583	0.037*	
C12B	1.0594 (5)	1.1636 (4)	0.06094 (13)	0.0466 (7)	
H12A	1.1739	1.2156	0.0783	0.070*	
H12B	0.9588	1.2452	0.0410	0.070*	
H12C	1.1134	1.0945	0.0298	0.070*	

### Atomic displacement parameters (Å^2^)

**Table:**

	*U*^11^	*U*^22^	*U*^33^	*U*^12^	*U*^13^	*U*^23^
F1A	0.0641 (12)	0.0530 (11)	0.0554 (10)	0.0053 (9)	0.0081 (9)	0.0204 (8)
O1A	0.0349 (9)	0.0197 (7)	0.0393 (9)	0.0029 (6)	0.0068 (7)	−0.0007 (6)
O2A	0.0261 (8)	0.0356 (9)	0.0384 (9)	−0.0011 (7)	0.0009 (6)	−0.0149 (7)
O3A	0.0242 (7)	0.0300 (8)	0.0383 (8)	−0.0041 (6)	0.0013 (6)	−0.0117 (6)
N1A	0.0288 (9)	0.0183 (8)	0.0324 (9)	−0.0005 (7)	0.0039 (7)	−0.0003 (6)
C1A	0.0234 (9)	0.0184 (8)	0.0256 (9)	−0.0015 (7)	−0.0016 (7)	−0.0034 (7)
C2A	0.0303 (10)	0.0161 (8)	0.0302 (10)	−0.0043 (8)	0.0005 (8)	−0.0038 (7)
C3A	0.0266 (10)	0.0186 (9)	0.0265 (9)	−0.0041 (8)	0.0001 (7)	−0.0007 (7)
C4A	0.0237 (9)	0.0189 (9)	0.0283 (9)	−0.0047 (7)	0.0022 (7)	−0.0013 (7)
C5A	0.0280 (11)	0.0319 (12)	0.0382 (12)	−0.0088 (9)	−0.0004 (9)	−0.0038 (9)
C6A	0.0293 (10)	0.0216 (9)	0.0297 (10)	−0.0051 (8)	0.0033 (8)	−0.0043 (7)
C7A	0.0321 (11)	0.0267 (10)	0.0339 (11)	−0.0020 (9)	0.0025 (9)	−0.0014 (8)
C8A	0.0440 (13)	0.0257 (11)	0.0343 (11)	−0.0052 (10)	0.0028 (10)	0.0013 (8)
C9A	0.0435 (13)	0.0271 (11)	0.0371 (12)	−0.0141 (10)	0.0096 (10)	−0.0065 (9)
C10A	0.0326 (12)	0.0346 (13)	0.0500 (14)	−0.0060 (10)	0.0117 (10)	−0.0057 (10)
C11A	0.0297 (11)	0.0284 (11)	0.0419 (12)	0.0001 (9)	0.0045 (9)	−0.0008 (9)
C12A	0.0585 (18)	0.0435 (15)	0.0430 (14)	−0.0181 (14)	0.0184 (12)	−0.0042 (11)
F1B	0.0517 (10)	0.0758 (13)	0.0364 (8)	−0.0110 (9)	−0.0070 (7)	0.0112 (8)
O1B	0.0296 (8)	0.0180 (7)	0.0375 (8)	0.0016 (6)	0.0067 (6)	0.0003 (6)
O2B	0.0248 (8)	0.0406 (10)	0.0422 (9)	−0.0002 (7)	0.0010 (6)	−0.0179 (7)
O3B	0.0226 (7)	0.0340 (9)	0.0409 (9)	−0.0035 (7)	0.0012 (6)	−0.0128 (7)
N1B	0.0279 (9)	0.0180 (8)	0.0365 (9)	0.0020 (7)	0.0068 (7)	−0.0007 (7)
C1B	0.0204 (9)	0.0186 (8)	0.0257 (9)	−0.0022 (7)	−0.0005 (7)	−0.0030 (7)
C2B	0.0285 (10)	0.0147 (8)	0.0315 (10)	−0.0045 (7)	0.0014 (8)	−0.0022 (7)
C3B	0.0253 (9)	0.0186 (9)	0.0261 (9)	−0.0044 (7)	0.0011 (7)	0.0009 (7)
C4B	0.0225 (9)	0.0205 (9)	0.0266 (9)	−0.0030 (7)	0.0024 (7)	−0.0009 (7)
C5B	0.0267 (11)	0.0346 (12)	0.0347 (11)	−0.0075 (9)	0.0008 (9)	0.0011 (9)
C6B	0.0254 (10)	0.0198 (9)	0.0330 (10)	−0.0007 (8)	0.0066 (8)	−0.0039 (7)
C7B	0.0242 (10)	0.0325 (11)	0.0355 (11)	−0.0059 (9)	0.0028 (8)	−0.0062 (9)
C8B	0.0347 (12)	0.0347 (12)	0.0304 (11)	−0.0019 (10)	0.0005 (9)	−0.0022 (9)
C9B	0.0342 (12)	0.0278 (11)	0.0362 (11)	−0.0049 (9)	0.0100 (9)	−0.0066 (9)
C10B	0.0282 (11)	0.0374 (13)	0.0384 (12)	−0.0102 (10)	0.0048 (9)	−0.0085 (9)
C11B	0.0263 (10)	0.0321 (11)	0.0345 (11)	−0.0037 (9)	0.0001 (8)	−0.0041 (9)
C12B	0.0529 (16)	0.0426 (15)	0.0451 (14)	−0.0156 (13)	0.0182 (12)	−0.0012 (11)

### Geometric parameters (Å, º)

**Table:**

F1A—C8A	1.355 (3)	F1B—C8B	1.356 (3)
O1A—C1A	1.228 (2)	O1B—C1B	1.223 (2)
O2A—H2A	0.8200	O2B—H2B	0.8200
O2A—C4A	1.315 (2)	O2B—C4B	1.311 (2)
O3A—C4A	1.229 (3)	O3B—C4B	1.225 (2)
N1A—H1A	0.8600	N1B—H1B	0.8600
N1A—C1A	1.345 (3)	N1B—C1B	1.344 (2)
N1A—C6A	1.418 (3)	N1B—C6B	1.429 (3)
C1A—C2A	1.524 (3)	C1B—C2B	1.523 (3)
C2A—H2AA	0.9700	C2B—H2BA	0.9700
C2A—H2AB	0.9700	C2B—H2BB	0.9700
C2A—C3A	1.500 (3)	C2B—C3B	1.499 (3)
C3A—C4A	1.483 (3)	C3B—C4B	1.488 (3)
C3A—C5A	1.320 (3)	C3B—C5B	1.325 (3)
C5A—H5AA	0.97 (3)	C5B—H5BA	0.95 (3)
C5A—H5AB	0.97 (3)	C5B—H5BB	0.96 (3)
C6A—C7A	1.387 (3)	C6B—C7B	1.380 (3)
C6A—C11A	1.392 (3)	C6B—C11B	1.391 (3)
C7A—H7A	0.9300	C7B—H7B	0.9300
C7A—C8A	1.382 (3)	C7B—C8B	1.377 (3)
C8A—C9A	1.373 (4)	C8B—C9B	1.381 (3)
C9A—C10A	1.385 (4)	C9B—C10B	1.388 (3)
C9A—C12A	1.508 (3)	C9B—C12B	1.508 (3)
C10A—H10A	0.9300	C10B—H10B	0.9300
C10A—C11A	1.384 (3)	C10B—C11B	1.389 (3)
C11A—H11A	0.9300	C11B—H11B	0.9300
C12A—H12D	0.9600	C12B—H12A	0.9600
C12A—H12E	0.9600	C12B—H12B	0.9600
C12A—H12F	0.9600	C12B—H12C	0.9600
			
C4A—O2A—H2A	109.5	C4B—O2B—H2B	109.5
C1A—N1A—H1A	116.0	C1B—N1B—H1B	118.9
C1A—N1A—C6A	128.04 (17)	C1B—N1B—C6B	122.27 (17)
C6A—N1A—H1A	116.0	C6B—N1B—H1B	118.9
O1A—C1A—N1A	123.59 (19)	O1B—C1B—N1B	123.28 (18)
O1A—C1A—C2A	122.32 (18)	O1B—C1B—C2B	122.36 (17)
N1A—C1A—C2A	114.09 (17)	N1B—C1B—C2B	114.34 (17)
C1A—C2A—H2AA	109.1	C1B—C2B—H2BA	109.3
C1A—C2A—H2AB	109.1	C1B—C2B—H2BB	109.3
H2AA—C2A—H2AB	107.9	H2BA—C2B—H2BB	107.9
C3A—C2A—C1A	112.28 (16)	C3B—C2B—C1B	111.76 (16)
C3A—C2A—H2AA	109.1	C3B—C2B—H2BA	109.3
C3A—C2A—H2AB	109.1	C3B—C2B—H2BB	109.3
C4A—C3A—C2A	115.60 (18)	C4B—C3B—C2B	115.88 (18)
C5A—C3A—C2A	123.3 (2)	C5B—C3B—C2B	123.4 (2)
C5A—C3A—C4A	121.1 (2)	C5B—C3B—C4B	120.7 (2)
O2A—C4A—C3A	114.93 (18)	O2B—C4B—C3B	114.48 (18)
O3A—C4A—O2A	123.6 (2)	O3B—C4B—O2B	123.6 (2)
O3A—C4A—C3A	121.48 (19)	O3B—C4B—C3B	121.93 (18)
C3A—C5A—H5AA	122.7 (18)	C3B—C5B—H5BA	119.9 (17)
C3A—C5A—H5AB	122.1 (19)	C3B—C5B—H5BB	120.1 (18)
H5AA—C5A—H5AB	115 (3)	H5BA—C5B—H5BB	120 (2)
C7A—C6A—N1A	123.2 (2)	C7B—C6B—N1B	120.5 (2)
C7A—C6A—C11A	119.2 (2)	C7B—C6B—C11B	119.9 (2)
C11A—C6A—N1A	117.53 (19)	C11B—C6B—N1B	119.6 (2)
C6A—C7A—H7A	121.1	C6B—C7B—H7B	120.7
C8A—C7A—C6A	117.9 (2)	C8B—C7B—C6B	118.5 (2)
C8A—C7A—H7A	121.1	C8B—C7B—H7B	120.7
F1A—C8A—C7A	117.0 (2)	F1B—C8B—C7B	117.6 (2)
F1A—C8A—C9A	117.8 (2)	F1B—C8B—C9B	118.4 (2)
C9A—C8A—C7A	125.1 (2)	C7B—C8B—C9B	124.0 (2)
C8A—C9A—C10A	115.3 (2)	C8B—C9B—C10B	116.0 (2)
C8A—C9A—C12A	122.7 (2)	C8B—C9B—C12B	122.2 (2)
C10A—C9A—C12A	121.9 (2)	C10B—C9B—C12B	121.8 (2)
C9A—C10A—H10A	118.9	C9B—C10B—H10B	119.0
C11A—C10A—C9A	122.3 (2)	C9B—C10B—C11B	122.0 (2)
C11A—C10A—H10A	118.9	C11B—C10B—H10B	119.0
C6A—C11A—H11A	119.9	C6B—C11B—H11B	120.2
C10A—C11A—C6A	120.1 (2)	C10B—C11B—C6B	119.5 (2)
C10A—C11A—H11A	119.9	C10B—C11B—H11B	120.2
C9A—C12A—H12D	109.5	C9B—C12B—H12A	109.5
C9A—C12A—H12E	109.5	C9B—C12B—H12B	109.5
C9A—C12A—H12F	109.5	C9B—C12B—H12C	109.5
H12D—C12A—H12E	109.5	H12A—C12B—H12B	109.5
H12D—C12A—H12F	109.5	H12A—C12B—H12C	109.5
H12E—C12A—H12F	109.5	H12B—C12B—H12C	109.5
			
F1A—C8A—C9A—C10A	−180.0 (2)	F1B—C8B—C9B—C10B	179.3 (2)
F1A—C8A—C9A—C12A	0.7 (4)	F1B—C8B—C9B—C12B	−0.5 (4)
O1A—C1A—C2A—C3A	−15.6 (3)	O1B—C1B—C2B—C3B	−13.9 (3)
N1A—C1A—C2A—C3A	165.26 (18)	N1B—C1B—C2B—C3B	167.55 (19)
N1A—C6A—C7A—C8A	−175.9 (2)	N1B—C6B—C7B—C8B	−179.3 (2)
N1A—C6A—C11A—C10A	177.1 (2)	N1B—C6B—C11B—C10B	179.2 (2)
C1A—N1A—C6A—C7A	−22.6 (4)	C1B—N1B—C6B—C7B	−71.6 (3)
C1A—N1A—C6A—C11A	160.4 (2)	C1B—N1B—C6B—C11B	109.7 (2)
C1A—C2A—C3A—C4A	−69.2 (2)	C1B—C2B—C3B—C4B	−69.6 (2)
C1A—C2A—C3A—C5A	113.4 (2)	C1B—C2B—C3B—C5B	112.1 (2)
C2A—C3A—C4A—O2A	168.57 (17)	C2B—C3B—C4B—O2B	168.70 (17)
C2A—C3A—C4A—O3A	−11.3 (3)	C2B—C3B—C4B—O3B	−11.7 (3)
C5A—C3A—C4A—O2A	−14.0 (3)	C5B—C3B—C4B—O2B	−13.0 (3)
C5A—C3A—C4A—O3A	166.2 (2)	C5B—C3B—C4B—O3B	166.6 (2)
C6A—N1A—C1A—O1A	−5.3 (4)	C6B—N1B—C1B—O1B	−0.6 (3)
C6A—N1A—C1A—C2A	173.9 (2)	C6B—N1B—C1B—C2B	177.90 (19)
C6A—C7A—C8A—F1A	178.7 (2)	C6B—C7B—C8B—F1B	−179.3 (2)
C6A—C7A—C8A—C9A	−0.7 (4)	C6B—C7B—C8B—C9B	0.8 (4)
C7A—C6A—C11A—C10A	−0.1 (4)	C7B—C6B—C11B—C10B	0.5 (3)
C7A—C8A—C9A—C10A	−0.6 (4)	C7B—C8B—C9B—C10B	−0.7 (4)
C7A—C8A—C9A—C12A	−179.9 (3)	C7B—C8B—C9B—C12B	179.4 (2)
C8A—C9A—C10A—C11A	1.6 (4)	C8B—C9B—C10B—C11B	0.6 (4)
C9A—C10A—C11A—C6A	−1.3 (4)	C9B—C10B—C11B—C6B	−0.6 (4)
C11A—C6A—C7A—C8A	1.0 (4)	C11B—C6B—C7B—C8B	−0.6 (3)
C12A—C9A—C10A—C11A	−179.1 (3)	C12B—C9B—C10B—C11B	−179.5 (2)

### Hydrogen-bond geometry (Å, º)

**Table:**

*D*—H···*A*	*D*—H	H···*A*	*D*···*A*	*D*—H···*A*
O2*A*—H2*A*···O3*A*^i^	0.82	1.84	2.658 (2)	174
O2*B*—H2*B*···O3*B*^ii^	0.82	1.85	2.667 (2)	176
N1*A*—H1*A*···O1*B*^iii^	0.86	2.10	2.948 (2)	168
N1*B*—H1*B*···O1*A*^iv^	0.86	2.10	2.884 (2)	151

Symmetry codes: (i) −*x*, −*y*+1, −*z*+1; (ii) −*x*+1, −*y*+2, −*z*+1; (iii) *x*, *y*−1, *z*; (iv) *x*+1, *y*, *z*.
